# Molecular assessment of *Theileria equi* and *Babesia caballi* prevalence in horses and ticks on horses in southeastern France

**DOI:** 10.1007/s00436-022-07441-7

**Published:** 2022-02-07

**Authors:** Gloria Rocafort-Ferrer, Agnès Leblond, Aurélien Joulié, Magalie René-Martellet, Alain Sandoz, Valérie Poux, Sophie Pradier, Séverine Barry, Laurence Vial, Loïc Legrand

**Affiliations:** 1 Equine Department, Université de Lyon, VetAgro Sup, Campus Vétérinaire de Lyon, 69280 Marcy l’Etoile, France; 2 La Clinique du Cheval, Centre Hospitalier Vétérinaire Équin, 3910 Route de Launac, 31330 Grenade, France; 3 EPIA, UMR 0346, Epidemiologie des maladies animales et zoonotiques, INRAE, VetAgro Sup, 69280 Marcy l’Etoile, France; 4 École Nationale Vétérinaire de Toulouse - Université de Toulouse, 31300 Toulouse, France; 5 Laboratoire Chimie de L’Environnement, CNRS, UMR 7376, Aix Marseille Université, 13003 Marseille, France; 6 EPIA, UMR 0346, Epidemiologie des maladies animales et zoonotiques, INRAE, VetAgro Sup, 63122 Saint-Genès-Champanelle, France; 7 Clinique Vétérinaire Jolimont, 31000 Toulouse, France; 8 BIOS Department, CIRAD-INRAE Joint Research Unit ASTRE (Animals, Health, Territories, Risks, and Ecosystems), Campus International de Baillarguet, 34398Cedex 5 Montpellier, France; 9 LABÉO Frank Duncombe, 14280 Saint-Contest, France; 10 UNICAEN, BIOTARGEN, Normandie University, 14000 Caen, France; 11 UNICAEN ImpedanCEL, Normandie University, 14280 Saint-Contest, France

**Keywords:** *Theileria equi*, *Babesia caballi*, Piroplasmosis, Ticks, Horse, *Rhipicephalus*

## Abstract

Equine piroplasmosis (EP) is a tick-borne disease caused by *Babesia caballi* and *Theileria equi* that is potentially emerging in non-endemic countries. We conducted a descriptive study to investigate EP prevalence and spatial distribution in an endemic region: the Camargue and the Plain of La Crau in France. In spring 2015 and 2016, we carried out sampling at stables (total *n* = 46) with a history of horses presenting chronic fever or weight loss. Overall, we collected blood from 632 horses, which were also inspected for ticks; these horses had been housed in the target stables for at least 1 year. We obtained 585 ticks from these horses and described land use around the stables. Real-time PCR was employed to assess *T. equi* and *B. caballi* prevalence in the horses and in the ticks found on the horses. For the horses, *T. equi* and *B. caballi* prevalence was 68.6% and 6.3%, respectively. For the ticks found on the horses, prevalence was 28.8% for *T. equi* and 0.85% for *B. caballi.* The most common tick species were, in order of frequency, *Rhipicephalus bursa*, *R. sanguineus sl.*, *Hyalomma marginatum*, *Haemaphysalis punctata*, and *Dermacentor* sp. Horses bearing *Rhipicephalus* ticks occurred in wetter zones, closer to agricultural areas, permanent crops, and ditches, as well as in drier zones, in the more northern countryside. Compared to horses bearing *R. bursa*, horses bearing *R. sanguineus sl.* more frequently occurred near the Rhone River. Prevalence of *T. equi* in the ticks was as follows: *Hyalomma marginatum* (43%), *Dermacentor* sp. (40%), *R. bursa* (33%), *R. sanguineus sl.* (19%), and *Haemaphysalis punctata* (17%). In contrast, *B. caballi* only occurred in *Dermacentor* sp. (20%) and *R. bursa* (1%).

**Supplementary Information:**

The online version contains supplementary material available at 10.1007/s00436-022-07441-7.

## Introduction

Equine piroplasmosis (EP) is a tick-borne disease caused by haemoprotozoan parasites, most commonly the piroplasms *Babesia caballi* and *Theileria equi*. Recently, researchers identified another piroplasm, *Theileria haneyi*, that may also potentially serve as an agent of EP (Knowles et al. 2018; Sears et al. 2019). In horses, EP can present as peracute, acute, subacute, or chronic disease. Common clinical signs include pale mucosa, limb oedemas, jaundice, and fever. *Theileria equi* infections may cause exercise intolerance in sport horses, which can mean substantially decreased earnings in the case of race horses industry (Padalino et al. 2019; El-Sherif et al. 2019).

It can be difficult to clinically assess chronic EP. Therefore, it is necessary to conduct several diagnostic tests and exclude the possibility of other infectious and non-infectious diseases to arrive at a final diagnosis and initiate appropriate treatment (Padalino et al. 2019). Moreover, EP symptoms are not highly specific; instead, they resemble those of other tick-borne diseases, such as anaplasmosis or Lyme disease (Zobba et al. 2008; Laus et al. 2013; Camino et al. 2019). The main difference between infection with *B. caballi* versus *T. equi* is that horses infected with *T. equi* can remain long-life carriers (Onyiche et al. 2019; Tirosh-Levy et al. 2020). Furthermore, the life cycles of these parasites are not exactly the same, since *B. caballi* exclusively infects red blood cells, while *T. equi* can also infect circulating leukocytes (Onyiche et al. 2019). These differences can potentially explain the contrasting responses of horses to treatments.

EP is endemic in tropical areas, subtropical areas, and some temperate areas, like those found in France. The disease is not endemic in Canada, New Zealand, Japan, the USA, Australia, or Singapore (Onyiche et al. 2019). Consequently, the international travel of horses is strictly monitored. Regulations in non-endemic countries can lead to seropositive horses being barred entry, resulting in significant economic losses (Guidi et al. 2015; Camino et al. 2019; Seo et al. 2020).

Despite its high levels of vigilance, the USA has already experienced two EP outbreaks. One outbreak occurred in Florida: it was iatrogenic in origin, resulting from shared needles and blood transfusions (Short et al. 2012). Twenty horses became infected with *T. equi*, and most were euthanised or exported to US federal research facilities. The other outbreak occurred naturally in Texas (Scoles et al. 2011). It was mediated by the tick *Amblyomma cajennense* and represented the first time this species had been observed to transmit *T. equi*. Up until that point, only one tick species, *Dermacentor variabilis*, had been found to act as a competent vector of EP in the USA.

In Europe, the main vectors of EP are ticks from the genera *Rhipicephalus*, *Dermacentor*, and *Hyalomma* (Onyiche et al. 2019). *Haemaphysalis* species can also act as competent vectors (Scoles and Ueti 2015). *Ixodes* species do not appear to have this ability.

The environmental persistence of ticks is mainly influenced by factors such as climatic conditions changing spatially but also according to seasons, as well as tick reproductive capabilities, and host presence (i.e., livestock and wild animals). In some areas, the persistence of tick-borne pathogens (TBPs) is largely mediated by tick density in the environment and specific transmission pathways (Harrison et al. 2011; Léger et al. 2013; Grech-Angelini et al. 2020).

In endemic areas, for the sake of monitoring and prevention, it is important to identify which vectors are better able to transmit TBPs to horses and to determine their geographical and habitat distributions. Moreover, EP and other diseases caused by TBPs have the potential to become emerging infectious diseases in non-endemic areas. Global changes, including climate change, are significantly boosting epidemiological risks by promoting the spread of vectors; indeed, a change is already being seen in the potential distribution of *Hyalomma marginatum* in southern France (Gray et al. 2009; Vial et al. 2016; Mysterud et al. 2017; Jourdain and Paty 2019; Paz 2020).

Our study was performed in the Camargue and on the Plain of La Crau, which form a region located in southeastern France (Supplementary File 1). This area has a Mediterranean climate, characterised by moderate temperatures and humidity, and contains a diversity of habitats (e.g., dry areas irrigated by canals, ditches, and wetlands) (Jourdain et al. 2007; Leblond et al. 2007). Large numbers of horses live outdoors and thus come into frequent contact with humans, domesticated animals, and wild animals.

The ticks most commonly encountered along the French Mediterranean coast are *Rhipicephalus bursa*, *Rhipicephalus sanguineus sl.*, *Dermacentor reticulatus*, and *Dermacentor marginatus*. Recently, *H. marginatum* was also observed in southern France (Vial et al. 2016) in two habitat types: wet areas in the Camargue and drier areas in the countryside near Montpellier. In addition to vectoring the piroplasms that cause EP, this species can also transmit Crimean-Congo haemorrhagic fever, a potential emerging infectious viral disease in this region (Vial et al. 2016).

Past work has found that the Camargue is a hyperendemic area for EP, where the seroprevalence of *T. equi* and *B. caballi* infection in horses has been estimated at 58% and 12.9%, respectively (Guidi et al. 2015). Only a few studies have used PCR to assess the piroplasms’ prevalence in horses and/or ticks in Europe, including in the Mediterranean Basin; to date, such work has been carried out in Spain, the UK, Ireland, Tunisia, and Israel (Ros-García et al. 2013; Coultous et al. 2019, 2020; Camino et al. 2021; Tirosh-Levy et al. 2021). Two other studies conducted in Europe characterised the piroplasms’ prevalence in ticks sampled from different hosts, including horses, in Corsica (Grech-Angelini et al. 2020) and Italy (Iori et al. 2010). In addition, work was performed in Tunisia to evaluate piroplasm prevalence in ticks and horses with a view to exploring the local genetic heterogeneity underlying EP in the region (Ros-García et al. 2013).

Our study had two key objectives: (1) to describe the geographical and habitat distributions of ticks found on horses in the study region and (2) to assess the prevalence of *T. equi* and *B. caballi* in horses and in ticks found on horses using real-time PCR, a first for this region.

## Materials and methods

### Study region and horse selection

The study was performed in the Camargue, located in the Rhone Delta, and on the Plain of La Crau, located east of the same delta (Supplementary File 1). Sampling took place at a range of stables in April–May 2015 (*n* = 37 stables) and May–June 2016 (*n* = 39 stables). There was some overlap among the stables such that a total of 46 different stables (Camargue = 19 stables; Plain of La Crau = 27 stables) were sampled across the two study seasons (Supplementary File 1). Global positioning system (GPS) coordinates were obtained for all the sites. In collaboration with local practicing veterinarians, we selected stables where there was a history of horses presenting unexplained chronic fever or weight loss, which are potential clinical signs of EP.

Over the course of the study, blood samples were collected from 632 horses (*n* = 338 in 2015 and *n* = 294 in 2016), which were also checked for ticks. The sample size for each stable was defined based on the stable’s total number of horses. We used a method previously described elsewhere (Guidi et al. 2015; Desjardins et al. 2018). A mean of 9 and 8 horses were sampled per stable in 2015 and 2016, respectively. The minimum number of horses sampled was 2, and the maximum was 15. The horses were also at least 1 year old and had been housed within the stable for at least 1 year. The owners of all the horses signed an informed consent form that detailed the main results expected and that attested to the confidentiality of all the information collected.

### Blood sampling and tick collection

The blood samples were collected by drawing blood into EDTA tubes via jugular venipuncture. The EDTA tubes were kept in coolers for half the sampling day, at most, and were then immediately frozen at − 20 °C.

As noted above, each horse was examined for the presence of ticks. When they were present, we collected attached but not engorged ticks (number collected—mean: 4 and range: 1–8). Specific body regions were carefully inspected during this process, namely the chin, shoulders, pasterns, base of the ears, back, inguinal region, and base of the tail. All the ticks were kept alive during each sampling period. Morphological identification to genus or species levels, depending on the ticks, was carried out using the bibliographic reference in tick taxonomy published by Pérez-Eid (Perez-Eid 2007), and the ticks were then frozen at -80 °C.

### DNA extraction—horse blood and ticks

The ticks were washed and then placed in individual tubes with metal beads. First, we added 300 µL of Dulbecco’s modified Eagle medium (DMEM) and 30 µL of foetal calf serum to each tube. Next, the tube contents were crushed using a Precellys tissue homogeniser (Bertin Technologies, Montigny-le-Bretonneux, France), yielding a liquid aliquot for each tick.

To extract DNA from the aliquots, we used a NucleoSpin® Blood QuickPure Kit (Macherey–Nagel, Bethlehem, USA) for the horse blood and the engorged ticks and a NucleoSpin® Tissue Kit (Macherey–Nagel) for the non-engorged ticks. Both kits were used in accordance with the manufacturer’s instructions. The DNA extracts were then stored at − 20 °C until testing could be performed.

We verified that the extraction process had been successful using a real-time PCR assay to confirm the presence of the 18S gene, highly conserved among all tick species. A positive result indicated that tick DNA (and any accompanying pathogen DNA) had been obtained.

### PCR assays—*Theileria equi* and *Babesia caballi*

The presence of DNA from *T. equi* and *B. caballi* was detected using real-time PCR performed on all the horse blood and tick samples; the methodology used is described elsewhere (Kim et al. 2008; Bhoora et al. 2010). The PCR assays were performed by LABÉO (Caen, France) using the Premix Ex Taq™ Kit (Takara, Saint-Germain-en-Laye, France). In brief, after an initial 3-min step at 95 °C, the analyses were run on StepOnePlus Real-Time PCR Systems (Applied Biosystems, Thermo Fisher, Villebon-sur-Yvette, France). There were 45 cycles of denaturation at 95 °C for 5 s and primer annealing at 64 °C for 35 s. To ensure the validity of the assays, positive and negative controls (blanks) were run in parallel with each PCR.

## Results

The stables were mostly located near wetlands, urban and peri-urban areas, arable land under rice, heterogenous agricultural zones, pastures, and forests. The horses housed in the stables were used for different types of activities, such as sports and leisure, horse breeding, or support for cow breeding.

### Prevalence of *Theileria equi* and *Babesia caballi* in horses

**Fig. 1:**
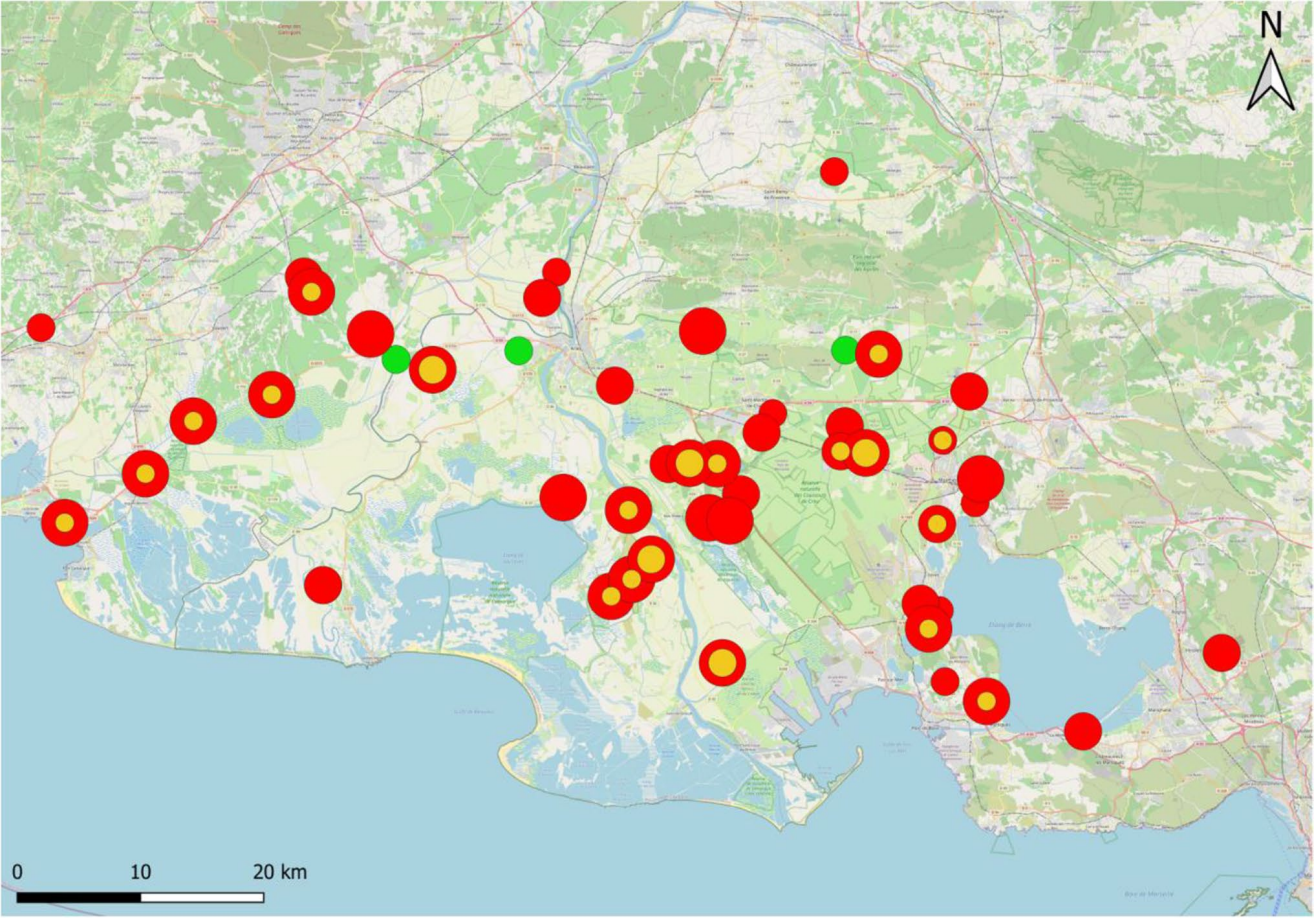
Geographical distribution of stables with horses positive for *T. equi* (red), positive for *B. caballi* (orange), or without any positive horses (green). Circle size is proportional to prevalence: small = 1–30%, intermediate = 31–60%, and large = 61–100% (map source: OpenStreetMap)

In the horses, the prevalence of *T. equi* and *B. caballi* across both years was 68.6% (434/632) and 6.3% (40/632), respectively. Overall piroplasm prevalence in the study region was 75% (474/632). Only 1.7% (11/632) of the horses were co-infected. Stables with piroplasm-positive horses were found across the entire study region (Fig. 1).

### Tick abundance and distribution

A total of 585 ticks were collected from the horses across the two study seasons (*n* = 150 ticks in 2015 and *n* = 435 ticks in 2016). Ticks were found in 23 of the 46 stables sampled (Supplementary File 2). In both years, the most commonly observed ticks were members of the genus *Rhipicephalus*, followed by members of *Hyalomma*, *Haemaphysalis*, and *Dermacentor* (Table 1).

**Table 1:** Species-specific numbers of ticks collected on the horses in 2015 and 2016

Ticks	2015	2016	Total
*Rhipicephalus sanguineus sl*	54	124	177
*Rhipicephalus bursa*	61	271	333
*Hyalomma marginatum*	7	40	47
*Haemaphysalis punctata*	23	0	23
*Dermacentor* sp.	5	0	5

**Fig. 2:**
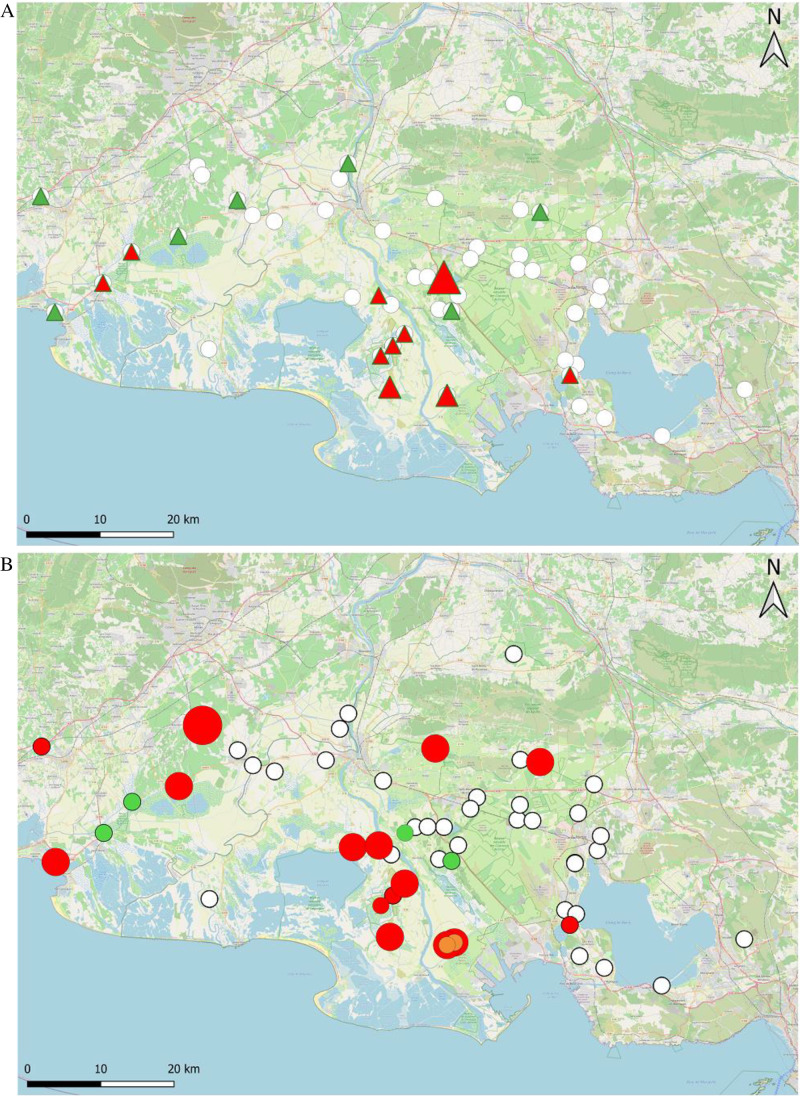
Geographical distribution of the stables where horses bearing *R. sanguineus sl.* (**A**) and *R. bursa* (**B**) were present. *Red*: stables where some ticks were positive for *T. equi*; *green*: stables where no ticks were positive for *T. equi*; *orange*: stables where some ticks were positive for *B. caballi*; *white:* stables where no *Rhipicephalus* ticks were collected. Circle and triangle size is proportional to piroplasm prevalence in the ticks (map source: OpenStreetMap)

Horses with *Rhipicephalus* ticks were found across the study region, generally in wetter zones, near agricultural areas, permanent cropland, and ditches, as well as in the drier, more northern countryside. However, they were rarely found in urban zones or coastal wetlands. The main difference in the occurrence of horses bearing *R. sanguineus sl.* versus horses bearing *R. bursa* was that the former were more common in stables near the Rhone, while the latter were found throughout the Camargue and the Plain of La Crau (Fig. 2). Horses with *Haemaphysalis punctata* were found in three stables in peri-urban areas, some near the coast and some in the countryside (Supplementary File 3). Most (21 out of 23) were housed in a single stable in the coastal wetlands east of the Rhone Delta. *Hyalomma marginatum* was found on horses located in just five stables, all near wetlands and farmlands (Supplementary File 4). *Dermacentor* sp. was found on horses in two different stables located 33 km apart in a wetter area. One stable was in a peri-urban area, while the other one was closer to wetlands (Supplementary File 4).

### *Theileria equi* and *Babesia caballi* prevalence in ticks

Most of the ticks (69%; 411/585) were negative for both *T. equi* and *B. caballi*. However, nearly a third (29.7%; 174/585) were positive for at least one of the piroplasms (Table 2). A very small percentage (0.51%; 3/585) were co-infected. Of the three co-infected ticks, one was *Dermacentor* sp., and two were *R. bursa* (Supplementary File 2).

**Table 2:** Tick species found on the horses and their prevalence of *Theileria equi* and *Babesia caballi*

Ticks	*T. equi* ^+^	*B. caballi* ^+^	No. stables	Prevalence (%) *T. equi*	Prevalence (%) *B. caballi*
*Rhipicephalus bursa* (*n* = 333)	110	4	19	33%	1%
*Rhipicephalus sanguineus sl.* (*n* = 177)	33	0	17	19%	0%
*Hyalomma marginatum* (*n* = 47)	20	0	5	43%	0%
*Haemaphysalis punctata* (*n* = 23)	4	0	3	17%	0%
*Dermacentor* sp. (*n* = 5)	2	1	2	40%	20%

#### *Theileria equi*

Overall, 28.8% (169/585) of ticks were positive for *T. equi*. Of these positive ticks, 84.6% (143/169) were *Rhipicephalus* species, 11.8% (20/169) were *H. marginatum*, 2.3% (4/169) were *H. punctata*, and 1.1% (2/169) were *Dermacentor.*

#### *Babesia caballi*

A very small number of ticks were positive for *B. caballi* (0.85%; 5/585); of the five total, four (80%) were *R. bursa*, and one (20%) was *Dermacentor.*

### Prevalence of *Theileria equi* and *Babesia caballi* in horses with ticks

Over the study period, around a quarter of the horses had at least one tick (23.4%; 148/632); 50 horses had ticks in 2015, and 98 horses had ticks in 2016 (Table 3). Among the horses with ticks, the vast majority were positive for at least one of the piroplasms (91.2%; 135/148).

**Table 3:** Prevalence of *Theileria equi* and *Babesia caballi* for different categories of horses

Category	Number of horses	Prevalence (%) *T. equi* ^+^	Prevalence (%) *B. caballi* ^+^
*All horses*	632	68.6%	6.3%
*Horses with ticks*	148	90.5%	9.4%
*Positive horses with positive ticks*	89	66%	7%

#### *Theileria equi*

Among the horses with ticks, the vast majority were positive for *T. equi* (90.5%; 134/148). Of these positive horses, two-thirds were carrying ticks that were also positive for *T. equi* (66%; 88/134); the full range of tick species was represented among them (102 *R. bursa*, 34 *R. sanguineus sl.*, 2 *H. punctata*, 22 *H. marginatum*, and 2 *Dermacentor*).

Of the tick-carrying horses that were negative for *T. equi* (8%; 12/148), five bore a total of nine ticks that were positive for the piroplasm (7 *R. bursa* and 2 *R. sanguineus sl*.).

#### *Babesia caballi*

Among the horses with ticks, 9.4% (14/148) were positive for *B. caballi*. One of these 14 positive horses was carrying two ticks that were also positive for *B. caballi* (a *Dermacentor* and a *R. bursa*). The rest of these horses (13/14) bore ticks that were negative for *B. caballi*. Four horses that were negative for *B. caballi* each bore 1 tick (all = *R. bursa*) that was positive for *B. caballi*.

## Discussion

This study is the first epidemiological research to use real-time PCR to characterise the prevalence of *T. equi* and *B. caballi* in horses and ticks found on horses in the Camargue and on the Plain of La Crau in southeastern France. We found that, in horses, *T. equi* prevalence was 68.6%, and *B. caballi* prevalence was 6.3%. In the ticks found on horses, *T. equi* prevalence was 28.8%, and *B. caballi* prevalence was 0.85%.

It is important to acknowledge that a few different factors could have influenced our results. First, we did not randomly choose the stables at which we sampled. Before beginning the study, we collaborated with practicing veterinarians to identify stables in which there had been cases of unexplained fever or weight loss. This approach could have somewhat biased our results since we did not sample from within the entire, perhaps more healthy horse population in the study region. That said, the stables’ geographical distribution was fairly homogenous, and we sampled horses living in different types of habitats. Second, our tick sampling procedures could have affected our findings. Notably, two new people were added to the sampling team in 2016. Third, sampling took place during different months across the 2 years. This fact could have influenced tick numbers since the different genera have different peaks of activity. These latter two reasons could partly explain the difference in tick numbers between 2015 and 2016. Fourth, tick numbers were likely to have been affected by any antiparasitic treatments. Half of the stables at which we sampled frequently perform such treatments to prevent tick infestations. Fifth, we classified the ticks based on morphology, not genetic analysis. While this approach could have resulted in some taxonomic errors, we believe that the potential for bias was minimal since most of the species collected display very distinct morphological characteristics. In addition, the ticks were identified by taxonomic experts. Sixth, we acknowledge that we were describing the habitats associated with ticks found on horses. While collecting ticks within the environment could have added complementary information to our study, we are not convinced it would have dramatically affected our findings. Indeed, we were working with Camargue horses, which live in “manades” and do not move among habitats. Since ticks cannot move freely over long distances by themselves, we feel confident that we are indirectly describing the habitats in which the ticks would be found even if they had been sampled within the environment.

In our study, we have confirmed that the Camargue and the Plain of La Crau in southeastern France form a region in which EP is highly endemic. High piroplasm prevalence was previously observed in the Camargue by researchers who used a complement fixation technique (Guidi et al. 2015). Their work found that 58% and 12.9% of horses were seropositive for *T. equi* and *B. caballi*, respectively. These prevalence levels are higher than those seen in other endemic countries, such as Spain, where recent PCR-based estimates of prevalence in horses were 29% for *T. equi* and 1.8% for *B. caballi* (Camino et al. 2021). These findings contrast with those from the UK, a non-endemic country, where prevalence was very low—0.8% for *T. equi* and 0% for *B. caballi* (Coultous et al. 2019). In general, compared to serological analysis, real-time PCR provides more specific but less sensitive assessments of prevalence (Tirosh-Levy et al. 2021). It is interesting to note that prevalence was higher for horses found to have ticks at the time of sampling (*T. equi*: 90.5%, *B. caballi*: 9.4%). Most of the horses in the study region are Camargue horses, and they live under semi-natural conditions. In addition, local environmental conditions mean they are more likely to be exposed to ticks and thus infected with piroplasms.

We found that the horses in the study region were most commonly carrying ticks in the genus *Rhipicephalus* (*R. bursa* and *R. sanguineus sl*.). Tick-bearing horses were present in wet and dry areas along the coast and in the countryside. These results concur with those from previous studies in the same region (René 2013; Chastagner et al. 2013). Both *R. bursa* and *R. sanguineus sl.* display similar distribution patterns in Europe and are mainly found in the Mediterranean Basin (Estrada-Peña et al. 2017). Horses are not the main hosts for these species. Instead, cattle are the principal host for *R. bursa*, whereas *R. sanguineus sl.* is primarily hosted by several species, including dogs and rabbits (René 2013; Estrada-Peña et al. 2017). Because horses in this region live alongside or graze with bulls, they are more likely to become infested with these ticks. The second most common tick we found on horses was *H. marginatum.* Its presence in southeastern France had been previously described (Vial et al. 2016). In contrast to what we observed, *H. marginatum* is the main tick found in other Mediterranean regions, including Corsica (Grech-Angelini et al. 2020), Tunisia (Ros-García et al. 2013), and Israel (Tirosh-Levy et al. 2021). However, this tick is described as a xerophilic species and environmental conditions in Camargue with predominant wet habitats may not be as much as suitable. We rarely observed ticks on horses belonging to the genera *Haemaphysalis* and *Dermacentor*, which fits with most past work (Iori et al. 2010; Ros-García et al. 2013; Tirosh-Levy et al. 2018). For *Dermacentor*, the period of work is not favourable as its activity period is mainly in autumn and at the beginning of spring. Finally, we did not observe any horses carrying *Ixodes ricinus*, even though it is the main tick species found in France and most of Europe (Estrada-Peña et al. 2017). This result is not necessarily surprising given that our study region was in the Mediterranean region. *Ixodes* ticks do not tolerate dry temperatures very well, and they need to live under conditions of high humidity (relative humidity > 80–85%) and along forest edges (Estrada-Peña et al. 2017).

We wish to underscore that detecting piroplasm DNA in ticks found on horses does not mean that the ticks are acting as vectors. It seems likely that horses with positive engorged ticks were responsible for infecting the ticks. However, it cannot be concluded that the ticks will be able to transmit the pathogen themselves. Our study is descriptive in nature: it has estimated the prevalence of equine piroplasmosis in horses and in ticks on horses. However, it did not seek to ascertain which ticks act as the main EP vectors in the region. That research will be pursued in other studies.

In this study, *T. equi* prevalence was highest in *H. marginatum* (43%). This finding concurs with others obtained in the Mediterranean Basin (Iori et al. 2010; Ros-García et al. 2013), except in the case of Corsica (Grech-Angelini et al. 2020). This result is of special interest since *H. marginatum* is an emergent tick species in the region (Vial et al. 2016), and its geographical distribution in southern France seems to be expanding. It raises an important question: will EP prevalence climb in this region if the tick continues to increase its range? While *Rhipicephalus* ticks were the most common, they did not have the highest *T. equi* prevalence (33% and 19% for *R. bursa* and *R. sanguineus sl*., respectively). The only previous study to observe *Rhipicephalus* ticks infected with *T. equi* in the Mediterranean Basin was carried out in Corsica (Grech-Angelini et al. 2020).

We found that *H. punctata* had the lowest *T. equi* prevalence (17%). This species has rarely been observed in the Mediterranean; however, as mentioned above, it was found in Israel (Tirosh-Levy et al. 2018). It remains unknown how well it can vector *T. equi*. While it has been described as a potential vector (Scoles and Ueti 2015), its prevalence of *T. equi* had never been assessed in the Mediterranean Basin until now. *Dermacentor* sp. was also uncommon in the study region, but the species had a relatively high *T. equi* prevalence (~ 40%).

With regard to *B. caballi*, prevalence was highest in *Dermacentor* sp. (20%). This tick has been described as a potential vector for *B. caballi* in Europe (Scoles and Ueti 2015). However, the prevalence of *B. caballi* in this species had never been assessed in the Mediterranean Basin. We observed that another tick, *R. bursa*, was also infected with *B. caballi* (1%). In contrast, no *H. marginatum* was positive for the pathogen. This result does not match what was found by Ros-Garcia and colleagues, who discovered that *H. marginatum* could be infected with *B. caballi* (2/97 ticks; Ros-García et al. 2013).

## Conclusion

This study is the first to use real-time PCR to estimate the prevalence of EP-causing piroplasms in horses and ticks found on horses in the Camargue and on Plain of La Crau in southeastern France. We found that *T. equi* appears to be highly endemic in our study region. We observed four tick genera (and five tick species) over the course of our sampling; *Rhipicephalu*s (*R. bursa*) was the most common*.* All five tick species were found to be infected with *T. equi*. In contrast, *B. caballi* was only found in *Dermacentor* sp. and *R. bursa.* The tick with the highest prevalence of *T. equi* was *H. marginatum*, at a similar level than observed in *Dermacentor*, a confirmed EP tick vector. The results of this study may have important implications for understanding, monitoring, and controlling EP, a disease that causes major economic losses in the horse industry. While our work was performed in a specific part of France, it helps fill the current gap of basic studies characterising the prevalence of EP pathogens in horses and ticks. In this regard, our findings may have broader implications for understanding disease dynamics across the European Mediterranean.

## Supplementary Information

Below is the link to the electronic supplementary material.

Supplementary file1 (DOCX 10324 KB)

## Data Availability

Not applicable.
