# Supplementary material for: Molecular assessment of Theileria equi and Babesia caballi prevalence in horses and ticks on horses in southeastern France
Source: Parasitol Res. 2022 Feb 7;121(3):999–1008. doi: 10.1007/s00436-022-07441-7 (PMC8858311; doi:10.1007/s00436-022-07441-7)
Supplement: Supplementary file 1 — Supplementary file1 (DOCX 10324 KB) [file 436_2022_7441_MOESM1_ESM.docx]

**SUPPLEMENTARY DATA**

**
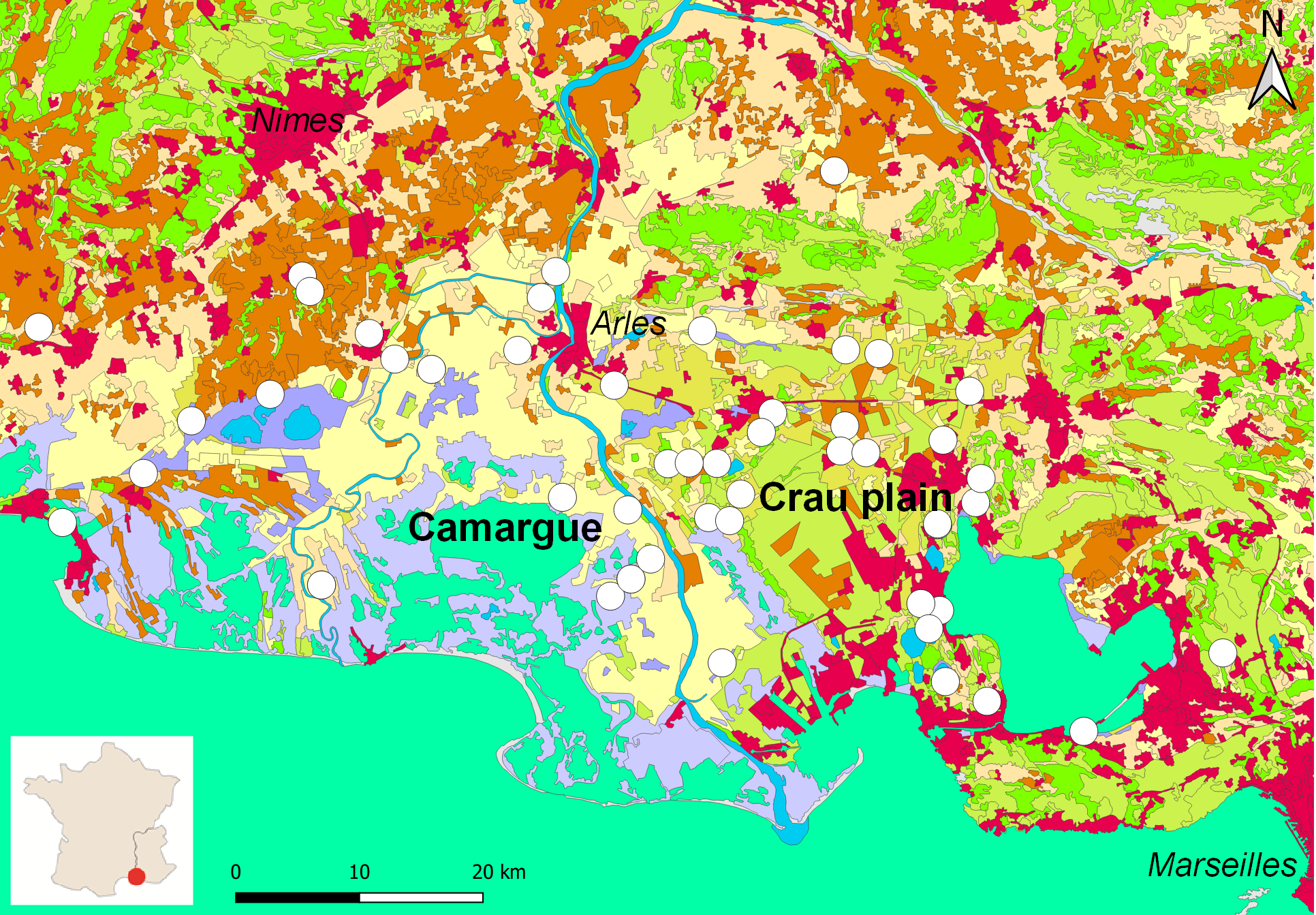
**

**Urban fabric**

**Permanent crops**

**Inland wetlands**

**Inland waters**

**Marine waters**

**Forests**

**Scrub and/or herbaceous vegetation associations**

**Pastures**

**Arable land**

**LEGEND**

**Supplementary file 1:** Geographical distribution of the stables where sampling took place (white circles) overlaid on a map showing the study region’s different habitat types (Source: European Union—SOeS, CORINE Land Cover, 2012). The map’s colour coding system is below:


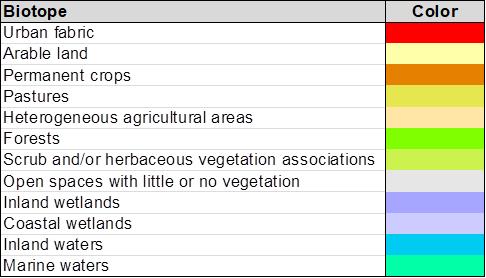


| **STABLES** | **N HORSES** | **HORSES *T. equi*+** | **HORSES**  ***B. caballi +*** | **HORSES+ TO BOTH PIROPLASMS** | **N TICKS \| total** | | | **TICKS *T. equi* + \| total** | | | **TICKS *B.caballi*+** |
| --- | --- | --- | --- | --- | --- | --- | --- | --- | --- | --- | --- |
| **1** | 27 | 27 | 3 | 3 | *R.bursa* (14)  *R.sanguineus sl* (37) | 51 | | *R.bursa* (4)  *R.sanguineus* (5) | 9 | | 0 |
| **2** | 19 | 19 | 0 | 0 | *R.bursa* (10)  *R.sanguineus sl* (11) | 21 | | *R.bursa* (1)  *R.sanguineus* (2) | 3 | |  |
| **3** | 18 | 16 | 2 | 2 | *H.punctata* (1)  *H.marginatum* (1)  *R.bursa* (21)  *R.sanguineus sl* (28) | 51 | | *H.punctata* (0)  *H.marginatum* (0)  *R.bursa* (11)  *R.sanguineus sl* (5) | 16 | | 0 |
| **4** | 18 | 18 | 1 | 1 | *R.bursa* (11)  *R.sanguineus sl* (14) | 25 | | R.bursa (4)  R.sanguineus sl (6) | 10 | |  |
| **5** | 26 | 26 | 8 | 8 | *Dermacentor sp* (3)  *R.bursa* (5)  R.sanguineus sl (16) | 24 | | *Dermacentor sp* (0)  *R. bursa* (2)  *R. sanguineus sl* (3) | 5 | |  |
| **6** | 20 | 20 | 2 | 2 | *R.bursa* (4) | | | 0 | 0 | | 0 |
| **7** | 18 | 13 | 1 | 1 | *R.bursa* (1) | | | 1 | 1 | | 0 |
| **8** | 19 | 19 | 0 | 0 | *R.bursa* (30) | | | 10 | 10 | | 0 |
| **9** | 2 | 2 | 0 | 0 | *R.sanguineus sl* (2) | | | 0 | 0 | | 0 |
| **10** | 2 | 1 | 0 | 0 | *R.bursa* (1) | | | 0 | 0 | | 0 |
| **11** | 8 | 6 | 1 | 1 | *R.bursa* (1) | | | 1 | 1 | | 0 |
| **12** | 7 | 7 | 2 | 2 | *R.bursa* (16) | | | 7 | 7 | | 2 |
| **13** | 9 | 9 | 2 | 2 | *H.punctata* (21)  *R.bursa* (7)  *R.sanguineus sl* (4) | | 32 | *H.punctata* (3)  *R.bursa* (0)  *R.sanguineus sl* (0) | 3 | | 0 |
| **14** | - | - | - | - | *R.sanguineus sl* (9) | | | 0 | 0 | | 0 |
| **15** | 6 | 6 | 0 | 0 | *R.bursa* (4) | | | 2 | 2 | | 0 |
| **16** | 20 | 20 | 3 | 3 | *Dermacentor sp* (2)  *H.punctata* (1)  *R.bursa* (34)  *R.sanguineus sl* (19) | 56 | | *Dermacentor sp* (2)  *H.punctata* (1)  *R.bursa* (5)  *R.sanguineus sl* (3) | 11 | | *Dermacentor sp* (1)* |
| **17** | 36 | 36 | 1 | 1 | *H.marginatum* (34)  *R.bursa* (7)  *R.sanguineus sl* (8) | 49 | | *H.marginatum* (18)  *R.bursa* (0)  *R.sanguineus sl* (1) | 19 | | 0 |
| **18** | 16 | 13 | 3 | 3 | *H.marginatum* (4)  *R.bursa* (25)  *R.sanguineus sl* (2) | 31 | | *H.marginatum* (0)  *R.bursa* (9)  *R.sanguineus sl* (0) | 9 | | 0 |
| **19** | 12 | 12 | 1 | 1 | *R.bursa* (3)  *R.sanguineus sl* (4) | 7 | | *R.bursa* (0)  *R.sanguineus sl* (1) | 1 | | 0 |
| **20** | 20 | 9 | 1 | 1 | *R.bursa* (63)  *R.sanguineus sl* (3) | 66 | | *R.bursa* (20)  *R.sanguineus sl* (0) | 20 | | 0 |
| **21** | 20 | 19 | 5 | 5 | *R.bursa* (64)  *R.sanguineus sl* (17) | 81 | | *R.bursa* (30)  *R.sanguineus sl* (7) | 37 | | *R.bursa* (2)* |
| **22** | 20 | 20 | 2 | 2 | *H.marginatum* (6)  *R.bursa* (6)  *R.sanguineus sl* (2) | 14 | | *H.marginatum* (2)  *R.bursa* (2)  *R.sanguineus sl* (0) | 4 | | 0 |
| **23** | 20 | 4 | 0 | 0 | *H.marginatum* (2)  *R.bursa* (6)  *R.sanguineus sl* (1) | 9 | | *H.marginatum* (0)  *R.bursa* (1)  *R.sanguineus sl* (0) | 1 | | 0 |
| **585** | | | **169** | | **5** | |  |  |  |  |  |

**Supplementary File 2:** Summary of data collected in the 23 stables in Camargue and Plain of la Crau where ticks were found on horses. The PCR results for the horses and the ticks found on the horses are included, as are the identities of the tick species collected. *****Ticks co-infected with *B. caballi and T. equi*

**
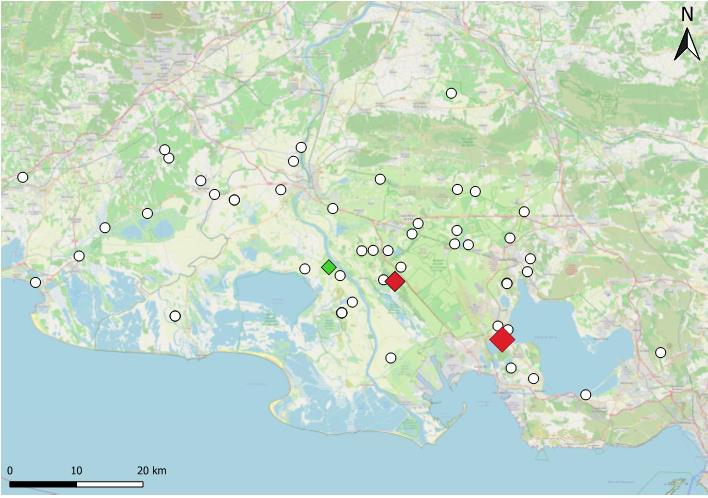
**

**Supplementary file 3:** Geographical distribution of the stables where *Haemaphysalis punctata* was present (diamonds). Red: stables where some ticks were positive for *T. equi*; green: stables where no ticks were positive for *T. equi;* white: stables without any *H. punctata*. Diamond’s size is proportional to piroplasm prevalence in the ticks (map source: OpenStreetMap).

**
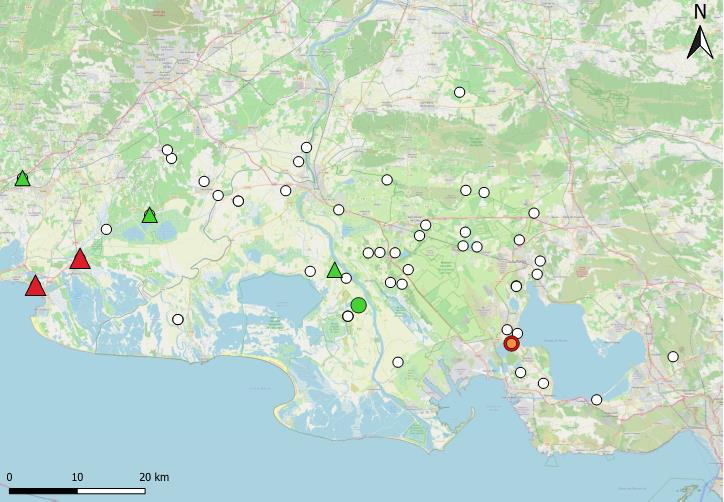
Supplementary file 4:** Geographical distribution of the stables where *Hyalomma marginatum* (triangles) and *Dermacentor sp* (circles) were present. Red: stables where some ticks were positive for *T. equi*; green: stables where no ticks were positive for *T. equi*; orange: stables where some ticks were positive for *B. caballi;* white: stables without any *H. marginatum* and/or *Dermacentor sp* Circle and triangle size is proportional to piroplasm prevalence in the ticks (map source: OpenStreetMap).
